# Colorectal polyps and polyposis syndromes

**DOI:** 10.1093/gastro/got041

**Published:** 2014-01-23

**Authors:** Noam Shussman, Steven D. Wexner

**Affiliations:** Department of Colorectal Surgery, Cleveland Clinic Florida, Weston, FL, USA

**Keywords:** colonic polyps, polyposis, screening

## Abstract

A polyp is defined as any mass protruding into the lumen of a hollow viscus. Colorectal polyps may be classified by their macroscopic appearance as sessile (flat, arising directly from the mucosal layer) or pedunculated (extending from the mucosa through a fibrovascular stalk). Colorectal polyps may also be histologically classified as neoplastic or as non-neoplastic (hyperplastic, hamartomatous, or inflammatory). The neoplastic polyps are of primary importance because they harbor a malignant potential, which represents a stage in the development of colorectal cancer. For this reason, it is essential to identify these polyps at a sufficiently early stage, when a simple outpatient procedure to remove them can interrupt the development of colorectal cancer and prevent disease and death. When invasive carcinoma arises in a polyp, careful consideration must be given to ensuring the adequacy of treatment. Although most neoplastic polyps do not evolve into cancer, it is well accepted that the majority of colorectal carcinomas evolve from adenomatous polyps; the sequence of events leading to this transformation is referred to as the adenoma-to-carcinoma sequence.

The presence of a systemic process that promotes the development of multiple gastro-intestinal polyps is termed ‘polyposis*’*. Hereditary gastro-intestinal polyposis syndromes account for approximately 1% of all cases of colorectal cancer and are associated with a broad spectrum of extra-colonic tumors. Early detection and accurate classification of these syndromes are essential, in order to initiate a surveillance program for the early detection of cancer. Several polyposis syndromes have been described, each having its own genetic basis and characteristic polyp distribution, clinical presentation, and malignancy risk.

Diagnostic modalities and treatment options for neoplastic polyps—as well as the most prevalent polyposis syndromes—are reviewed below.

## COLORECTAL POLYPS

‘Polyp’ is a term derived from the Greek word *polypous*, which means ‘morbid lump.’ Generally, this term describes any mass protruding into the lumen of a hollow vessel, anywhere in the gastro-intestinal, genito-urinary or respiratory tracts. Usually, polyps arise from the mucosal layer of these organs, although some submucosal pathologies may cause mucosal protrusion into the lumen and resemble mucosal polyps. Not all polyps necessarily exhibit neoplastic behavior.

Colorectal polyps may be histologically classified as neoplastic, hyperplastic, hamartomatous, or inflammatory. The neoplastic polyps are of primary importance because they harbor a malignant potential, which represents a stage in the development of colorectal cancer. For this reason, it is essential to identify these polyps at a sufficiently early stage, when a simple office procedure to remove them can stop the development of colorectal cancer and prevent disease and death.

**Figure 1.**
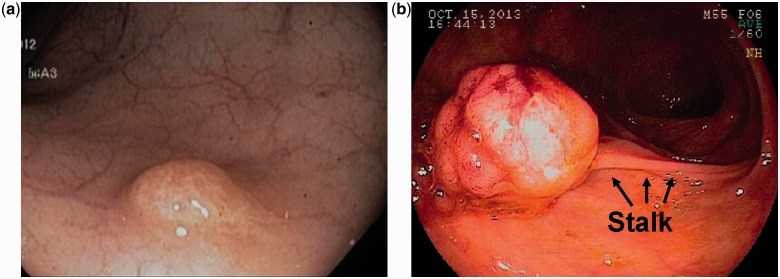
Colonoscopic view of sessile (1a) and pedunculated (1b) polyps.

Colorectal polyps may be classified by their colonoscopic appearance as sessile (flat, arising directly from the mucosal layer) or pedunculated (extending from the mucosa through a fibrovascular stalk) (Figure 1).

## NEOPLASTIC COLORECTAL POLYPS

Neoplastic (adenomatous) colorectal polyps are benign tumors that originate from the mucus-secreting colonic epithelial cells. Adenomatous polyps are common, especially in western countries, occurring, in the United States, in 20–40% of screening colonoscopies in people older than 50 years of age. One recent series reported that adenoma rates depend on age and gender [1]. In people younger than 50 years of age, 12% of women and 24% of men were found to have an adenoma on a screening colonoscopy. In women and men older than 80 years, the rates increased to 27% and 40%, respectively [1]. A recent population-based study found that at least one polyp was detected in 34.3% of asymptomatic patients undergoing a screening colonoscopy [2]. In autopsy series, the prevalence is even higher and increases with age. One-third to one-half of patients found to have a colonic adenoma have a synchronous colonic lesion [3]. The factors contributing to the development of colonic adenomas are multiple and uncertain; however, it is well accepted that both genetic susceptibility and environmental factors play a role in this process. Smoking was shown to be a risk factor for the development of colonic polyps [4]; others include obesity, high intake of red meat and low intake of fiber and calcium [5, 6]. Conversely, the use of non-steroidal anti-inflammatory medications (NSAIDs) and of statins, has been shown to have a protective effect [5, 7]. Anatomically, adenomas may be found anywhere throughout the colon. Large adenomas—which are more prone to develop into carcinomas—are found in a distribution similar to that of carcinomas, with left colonic predominance. Clinically, most polyps are not symptomatic and are found on screening colonoscopies. Larger polyps may bleed or partially obstruct the colonic lumen; therefore, hematochezia (visible or occult) or obstructive symptoms such as abdominal pain, swelling, or change in bowel habits may prompt investigation. Secretory diarrhea and hypokalemia (McKittrick-Wheelock syndrome) may be rare clinical presentations of a villous adenoma.

**Figure 2.**
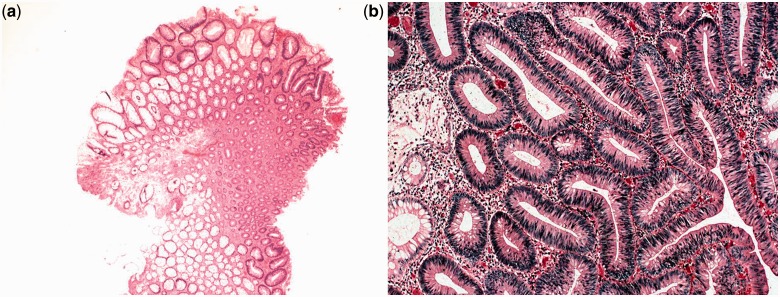
Tubular adenoma: low (×400, 2a) and high (×2000, 2b) power magnification views of a hematoxilin–eosin stain. The histological appearance is of branched tubular glands. Courtesy of Mariana Berho, MD.

**Figure 3.**
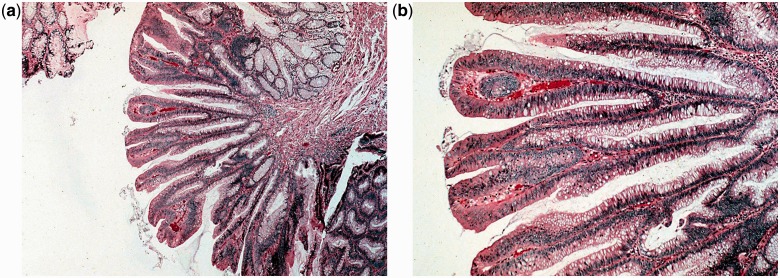
Villous adenoma: low (×400, 3a) and high (×2000, 3b) power magnification views of a hematoxilin–eosin stain. The histological appearance is of long finger-like projections. Courtesy of Mariana Berho, MD.

Adenomatous polyps are sub-classified by their histological appearance as tubular, villous, or tubule-villous adenomas. A tubular adenoma has the histological appearance of branched tubular glands (Figure 2). This is the most common histological subtype, constituting approximately 65–80% of all polyps removed. Tubular adenomas are most often pedunculated and generally harbor less atypia than villous adenomas do, although the degree of atypia is variable. Villous adenomas have long, finger-like projections on microscopy (Figure 3). Only 5–10% of neoplastic polyps are villous adenomas. Compared with tubular adenomas, villous adenomas are more commonly sessile and are more likely to have severe atypia or dysplasia. Tubulo-villous adenomas have elements of both of the cellular patterns that were previously discussed. Approximately 10–25% of polyps are tubulo-villous. The incidence of invasive carcinoma in a polyp is classically considered to depend on its size and histological type. The respective risks of carcinoma are less than 5% in a tubular adenoma smaller than 1 cm and may reach 50% in a large (>2 cm) villous adenoma. Tubulo-villous adenomas are at intermediate risk (22%). One recent study, however, did not identify size and histological sub-type as risk factors for development of cancer in polyps [8]. By definition, all types of adenomas exhibit some degree of dysplasia, meaning abnormal glandular architecture and damaged intracellular structures. The degree of dysplasia is diverse and, as it becomes more severe (high grade), the risk of malignancy increases. In fact, the term ‘high-grade dysplasia’ is synonymous with carcinoma *in situ*. The degree of dysplasia usually correlates with villous histology and polyp size.

### Adenoma-to-carcinoma sequence

Although most neoplastic polyps do not evolve to cancer, it is well accepted that the majority of colorectal carcinomas evolve from adenomatous polyps; the sequence of events leading to this transformation is referred to as the adenoma-to-carcinoma sequence.

The formation of a neoplastic process requires multiple cumulative genetic alterations that can be divided into three categories:

- Mutations in proto-oncogenes that induce their transformation into active oncogenes (these are genes that play a role in intracellular signal transduction and their activation causes abnormal transmission of growth regulatory signals).
- Mutations or deletions that reduce the activity of tumor suppressor genes.
- Mutations that cause damage to genes involved in DNA repair.

These three mechanisms contribute to uncontrolled cell proliferation, autonomous growth and, hence, tumor formation [9]. It is assumed that the neoplastic process starts along with the intracellular expression of the first genetic mutations and, as the genetic damage continues to accumulate, the neoplastic process becomes more advanced. A critical point in this process occurs when the neoplastic cells gain the ability to penetrate the basement membrane and to metastasize. This point defines the transformation of an adenoma to a carcinoma [10].

Several genes have been recognized as playing a role in the development of colorectal cancer via the adenoma-to-carcinoma sequence. These genes include the tumor suppressor genes APC, DCC, and p53; the proto-oncogenes K-RAS and MYC; and the DNA mismatch repair genes MLH1, MSH2, and MSH6. Each of these genes is believed to have a specific stage of tumor formation in which its de-activation (tumor suppressor gene) or activation (proto-oncogene) is critical. For example, the protein product of the p53 gene has a role in preventing damaged cells from going through the mitotic cycle and its loss is believed to mediate the conversion of an adenoma to a carcinoma [11]. It is worth mentioning that the loss of activity of a tumor suppressor gene requires mutation or deletion of both alleles [12]. A demonstration of this principle can be found in some inherited syndromes, where an inherited (germline) mutation in one tumor suppressor gene allele causes the individual to be susceptible to tumor formation; then a second (somatic) mutation is caused by environmental factors and induces damage to the other allele. Tumor formation is initiated at that time. Such individuals have the tendency to develop tumors at an early age [13]. An example of these inherited syndromes is familial adenomatous polyposis (FAP), in which a mutation in one of the APC tumor suppressor gene alleles causes susceptibility to colorectal cancer. This syndrome, along with other inherited polyposis syndromes, is discussed in further detail below.

The discussion on the adenoma to carcinoma adenoma-to-carcinoma sequence would not be complete without mentioning epigenetic alterations (including DNA methylation) and micro-environmental influences, but these are beyond the scope of this review. The exact pace at which an adenoma transforms into an invasive tumor is not known. It is estimated that in most cases it takes 5–10 years for a visible polyp to become a carcinoma.

## CARCINOMA IN POLYPS

As described earlier, adenomatous polyps should be considered to be precursors of cancer. The risk of a polyp harboring cancer is related to several factors. One of these—which is obvious upon its identification during colonoscopy—is its size. A recent study has shown that the risk of a polyp no larger than 5 mm harboring high-grade neoplasia is less than 1%, and the risk of its harboring cancer is negligible [14]. However, polyps larger than 5 mm in size were shown to have a 3% chance of harboring cancer and the chance increases with increasing polyp size. When invasive carcinoma arises in a polyp, careful consideration must be given to ensure the adequacy of treatment. Carcinoma confined to the *muscularis mucosae* does not metastasize, and complete excision of this type of polyp is adequate treatment. Nevertheless, penetration through the *muscularis mucosae* indicates that the tumor has gained the ability to metastasize; thus the adequacy of simple polypectomy is in question. Haggitt and colleagues have proposed a classification system for polyps containing cancer, that has become accepted for determining whether such a polyp requires a wider excision [15]. According to this system, all of the following criteria must be met in order for a complete polypectomy to be considered as an adequate treatment:

— The polyp must be pedunculated
— The tumor does not extend beyond the head or neck of the polyp (Haggitt's levels 1 and 2, respectively)
— The distance between the tumor edge and the margin of the specimen exceeds 2 mm
— The histology is favorable (not poorly differentiated and no lymphatic or vascular invasion)

However, if any one of the following situations occurs when a cancer-containing polyp is excised, polypectomy alone is not considered a safe enough strategy, due to the 10% or greater chance of lymph node metastasis:

— The polyp is sessile
— A pedunculated polyp is identified with invasion of the tumor to any part of its stalk beneath its neck or to the submucosa of the bowel wall beneath the stalk (Haggitt's levels 3 and 4, respectively)
— The distance between the tumor edge and the margin of the specimen is less than 2 mm
— The histology is poorly differentiated
— Lymphatic or vascular invasion is observed

If any of these situations occurs, surgical resection of the affected colonic segment according to oncologic principles is generally recommended. Such patients are often excellent candidates for laparoscopic colectomy.

### Diagnostic modalities for the diagnosis and evaluation of neoplastic polyps

As mentioned above, most colonic adenomas are asymptomatic. Due to the widespread character of this phenomenon and the risk of malignancy, many countries have introduced screening programs for early detection of colonic adenomas.

#### Fecal occult blood testing

(FOBT) may indicate bleeding from a colonic polyp. A positive FOBT due to bleeding from a polyp correlates to the polyp size and proximity to the rectum. Most small polyps will fail to result in a positive FOBT, although the test has a higher sensitivity for larger polyps and for carcinomas. For this reason, FOBT is a part of the screening algorithm for the early detection of colon cancer, despite its poor sensitivity for polyps.

#### Fecal immunochemical testing

(FIT or iFOBT) is a newer, more sensitive screening method than the traditional FOBT. It utilizes specific antibodies to the globin component of the hemoglobin. A recent study compared FIT against colonoscopy as a screening tool for both colorectal cancer and adenomas [16]. Even though FIT was shown to be as sensitive as colonoscopy in detection of cancer, advanced adenomas were detected in lower proportions using FIT, when compared with colonoscopy (0.9 vs 1.9%; odds ratio 2.30; *P < *0.001) and non-advanced adenoma detection rate was even lower (0.4 vs 4.2%; odds ratio 9.80; *P < *0.001).

#### Colonoscopy

is the ‘gold standard’ method of detecting intra-luminal colonic lesions. However, its sensitivity is not 100%. Several studies have demonstrated a variable ‘missed’ polyp rate. One systematic review included six studies covering a total of 465 patients who underwent two same-day colonoscopies. The ‘miss’ rate for polyps of any size was 22%, adenoma miss rate by size was 2.1% for adenomas ≥10 mm, 13% for adenomas 5–10 mm and 26% for adenomas 1–5 mm [17]. Other studies have shown similar results. Still, because most large villous polyps are distributed throughout the left colon, screening flexible sigmoidoscopy every five years, beginning at age 50, is recommended by the World Health Organization and others [18]. Another screening strategy, recommended by the American Cancer Society, is full colonoscopy every ten years, beginning at age 50 [19].

#### Colonoscopic spectroscopy

using near-infrared autofluorescence (NIR AF) was recently proposed as an adjunct for *in vivo* diagnosis of colonic ‘pre-cancer’ and cancer during clinical colonoscopic screening. This method was found to have a sensitivity and specificity of approximately 80% and 90%, respectively, for classification of benign, pre-cancer lesions and cancer in the colon [20]. This method, although promising, is still experimental and is not routinely used in clinical practice.

#### Narrow-band imaging

(NBI) is another new endoscopic imaging technique that highlights surface structures and superficial mucosal capillaries during colonoscopy. Even though disagreement exists regarding its effectiveness in increasing the colonoscopic view’s sensitivity, it has recently been shown to have a high sensitivity and specificity for differentiating neoplastic and non-neoplastic polyps [21, 22]. This modality has also not entered routine clinical practice.

#### Computed tomographic colonography

(also called ‘CT colonography’ or ‘virtual colonoscopy’) is another screening modality, which is suggested for patients who refuse colonoscopy. This modality uses computed tomography of an air-distended prepared colon. With an optimal colon preparation and an experienced radiologist reading the images, some reports indicate that the sensitivity of CT colonography for detecting polyps larger than 5 mm (which are believed to be clinically significant) exceeds 90% [23, 24]; however, other reports have noted lower sensitivity and pitfalls. Hence, the examination is mainly recommended for patients at increased risk associated with sedation for colonoscopy, or with difficult anatomy that challenges the successful completion of a full colonoscopy. As indicated above, other prerequisites for the successful use of CT colonography are optimal colon preparation, appropriate computers and software, and an experienced radiologist to interpret the acquired images [24]. It should be emphasized that CT colonography does not enable polypectomy and therefore, whenever CT colonography identifies polyps, sequential colonoscopy and polypectomy are indicated.

#### Magnetic resonance colonography

(MRC) is another diagnostic modality that is currently being evaluated. The rationale for using MRC is based on the relatively high radiation exposure during CT colonography [25]. A recent small-scale study has demonstrated a low sensitivity (despite a high specificity) for detecting large (>10 mm) polyps using MRC [26]. Therefore, evidence does not support MRC as a standard diagnostic modality for detecting colorectal polyps and this modality is not routinely used in clinical practice.

#### Capsule endoscopy

is a diagnostic modality that was originally developed to diagnose and evaluate small bowel lesions. Since the capsule passes through the prepared colon after traversing the ileo-cecal valve and continues to transmit images, it can also detect colonic lesions. A large cohort of patients (328) with suspected colonic lesions underwent a capsule endoscopy with dual camera capsule designed especially to evaluate the colon (PILLcam colon) and, immediately afterwards, had a colonoscopy. The sensitivity and specificity of the capsule endoscopy were shown to be inferior to colonoscopy [27]. Specifically the PILLcam colon was found to have sensitivities of 64% for the detection of polyps larger than 6 mm, 73% for the detection of advanced adenomas (larger than 1 cm, villous or containing high-grade dysplasia) and 74% for the detection of cancer. Hence, it is not recommended as a screening modality for the detection of colonic polyps or cancer.

#### Fecal DNA and antigen testing

is another futuristic modality expected to yield results within the next few decades [28]. Several technical advances have recently been seen to increase its accuracy, including use of a DNA preservative buffer with stool collection, DNA amplification methods and automated assays of several DNA markers [29]. A recently published multicenter, case-control study, compared colonoscopy with analysis of stool sample from 459 asymptomatic patients and 544 referred patients. The stool was analysed with an automated multi-target stool DNA assay to measure *β-*actin, mutant KRAS, aberrantly methylated BMP3 and NDRG4, and fecal hemoglobin. Stool DNA analysis identified individuals with colorectal cancer with 98% sensitivity and 90% specificity. Its sensitivity in respect of advanced adenomas was 57% and for high-grade dysplasia it was 83% [30]. In the future, should this modality prove to have even a higher positive predictive value for detecting adenoma or carcinoma, it might obviate the need for any invasive screening tests.

## TREATMENT OF NEOPLASTIC COLONIC POLYPS

### 

The definitive treatment of an adenomatous polyp is removal upon detection—although some debate exists regarding the need for these procedures on polyps smaller than 10 mm that are diagnosed with a non-invasive procedure, specifically CT colonography. An accepted approach to this issue is to resect any small (6–9 mm) polyps and to report and to periodically re-assess any diminutive (5 mm or smaller) polyps, due to the negligible malignant potential in the latter case [31].

#### Endoscopic forceps polypectomy

is one of the simplest maneuvers in the endoscopist’s armamentarium. It is a simple and efficient method of biopsying colonic lesions but has been shown to have a lower rate of histological clearance at polyps’ bases, compared with snare polypectomy (75.9 vs 93.2%; *P = *0.009 in a recent randomized trial) regardless of the polyps’ sizes [32].

#### Endoscopic snare polypectomy

is the preferred way to remove a polyp. During this procedure, a wire (the snare) is passed through the working channel of the endoscope to encircle the polyp at its base or ‘stalk’. Monopolar electric current may be carefully applied at the wire (‘hot snare’) while pulling the polyp. This procedure is simple for pedunculated polyps but can be quite challenging for sessile ones. A technique for making snare polypectomy feasible for sessile polyps is to inject saline into the submucosal layer beneath the polyp. The site of sessile polypectomy should be marked by injection of ink (tattooing) to guide follow-up colonoscopy and to facilitate identification of the involved bowel segment, should operative resection be necessary. The actual presence of the polyp is an indication for a complete colonoscopy to exclude synchronous lesions.

#### Endoscopic sub-mucosal dissection

(ESD) is a technique for resecting sessile polyps that are not amenable to snare polypectomy. During colonoscopy, the submucosal layer of the bowel wall beneath the polyp is injected with Saline-adrenaline and dissected from the underlying muscular layer. This method aims to remove the entire sessile polyp, along with healthy mucosal borders. This procedure is time-consuming and technically demanding; however, in skilled hands and in well-selected patient cases, it can obviate surgery. One recent series of endoscopic polypectomies for giant (>30 mm) pedunculated polyps showed a 100% success rate of *en bloc* resections using ESD for polyps that were deemed technically too difficult for a snare polypectomy [33].

#### Complications of polypectomy

include bleeding and perforation, the incidence of both being low [34, 35]. Bleeding may occur immediately after polypectomy or may be delayed. If the bleeding does not spontaneously cease, colonoscopy is indicated to secure hemostasis. The risk of perforation increases with the complexity of the procedure and is higher for sessile polyp excision (whether or not a submucosal dissection was performed). A micro-perforation that is evident only as extra-luminal air bubbles on CT, in a stable patient with no clinical signs of peritonitis, may be managed with bowel rest, antibiotics and observation. Signs of sepsis or peritonitis are indications for urgent surgical exploration via laparotomy or laparoscopy, in order to repair the damage or to resect the perforated bowel segment.

#### Surgery

is sometimes indicated for the treatment of polyps. Sessile lesions cannot always be completely excised by colonoscopy and such cases are an indication for segmental colectomy. Another indication for surgery is in cases of a pedunculated polyp, containing invasive carcinoma that extends into the polyp stalk, as was described in Haggitt's criteria [15]. Nevertheless, it is important to note that a partial colectomy for removing a polyp that is not amenable to colonoscopic resection is potentially a procedure for treating cancer. A recent large-scale study (750 patients) actually found that the incidence of cancer in patients undergoing colectomy for an irretrievable polyp is 17.7% [8]. Multivariate analysis identified two risk factors for the polyps harboring cancer: polyp location at the left colon and the presence of high grade dysplasia. Surprisingly, neither polyp size nor villous histology was found to be a risk factor. Given these data, surgery for irretrievable polyps should follow oncological guidelines, meaning anatomical resection, including the relevant mesentery containing the lymphatic basin with arterial high ligation. For rectal sessile polyps, transanal operative excision—using either simple transanal excision or transanal endoscopic microsurgery (TEM)—is preferred for two reasons: (i) it carries a higher probability of complete excision than does endoscopic snare excision and (ii) it produces an intact specimen that can be used to determine the need for further therapy.

#### Follow-up

is needed for patients who are found to have colonic polyps, due to the 30–40% likelihood of the appearance of metachronous lesions within three years. Therefore, following polypectomy, repeat colonoscopy at three-year intervals is indicated. If the repeat examination does not reveal metachronous lesions, continued follow-up is advised at five-year intervals [18]. If the primary (index) polyp contains high-grade dysplasia or carcinoma on histology, the chance of an early histologically significant metachronous lesion is high. Hence, in such a case the recommendation is for the first follow-up at one year.

There are no guidelines for the prevention of adenoma formation. Several studies have shown a slight but statistically significant lower prevalence of colorectal adenomas and cancers in people who consume NSAIDs, including aspirin [36, 37]. Nevertheless, due to risks of NSAID toxicity, there is no consensus regarding their use for this indication. No other strategies have been proven to prevent the development of colonic adenomas.

### Serrated adenomas

Up to recent years, colorectal polyps were traditionally classified as either adenomatous or non-neoplastic. In recent years, accumulating evidence points at another type of polyp, once believed to be a subset of hypeplastic polyp, which now is known to bear a malignant potential. These were given the name ‘serrated polyps’ due to their histological appearance (of serrated papillary infoldings into the crypts). Serrated polyps are a heterogeneous group of polyps—some of which were defined in 2003 as a subgroup—and are called ‘sessile serrated adenomas’ (SSAs). These tend to develop at the right colon and more often in women than in men [38]. SSAs have a characteristic flat and irregular endoscopic appearance and histological findings of extension of the serrations to the crypt base and of dilated L or inverted T-shaped crypts. They also appear to be associated with the microsatellite instability characteristic of defects in DNA repair mechanisms (mainly hypermethylation of the MLH1 gene), similar to DNA changes seen in sporadic microsatellite unstable cancers [38, 39]. Polyps of another subtype, called ‘traditional serrated adenomas’ (TSAs), tend to bear mutations at the BRAF gene and present a high rate of hypermethylation of multiple genes, a characteristic termed ‘CpG island methylator phenotype’ (CIMP) [38–42]. These changes are also similar to ones seen in sporadic microsatellite unstable cancers [38, 39]. Large (>10 mm) serrated polyps were also shown to be associated with synchronous advanced neoplasia [39, 43]; hence, it has now become accepted that a faster adenoma-to-carcinoma sequence exists, which is referred to as the ‘serrated adenoma pathway’. This pathway has recently been postulated to be responsible for the development of as much as 15–30% of colon cancers [38, 39, 42]; hence, even though once considered to be of low malignant potential, any serrated adenoma larger than 5 mm should be excised, with the incentive to completely remove it. If a serrated adenoma is not amenable to complete endoscopic polypectomy, segmental colectomy should be performed [44]. The recommended surveillance for patients found to have a serrated adenoma is the same as for patients found to have adenomatous polyps, suggesting repeat colonoscopy at three-year intervals [18, 38, 39].

### Dysplasia-associated lesion or mass

Dysplasia-associated lesion or mass (DALM) refers to a finding of a raised mucosal lesion in a patient with long-standing inflammatory bowel disease (IBD), mainly mucosal ulcerative colitis (MUC), in which dysplasia is found on histological examination. DALM can be indistinguishable macroscopically from an inflammatory pseudopolyp, aside from the fact that inflammatory pseudopolyps are usually not solitary. These lesions can resemble a ‘regular’ polyp or may be irregularly delineated, plaque-like, or irregularly elevated. The importance of DALMs is that they harbor a high risk of progression to cancer. The transition of a DALM to colorectal cancer is believed to be much faster than the classic adenoma-to-carcinoma sequence. The presence of a DALM is also considered to be a sign of a pre-cancerous condition of the entire colon, affected by the long-standing inflammatory condition; hence the finding of a DALM in a patient with MUC by itself is an indication for a total proctocolectomy, with or without reconstruction [45].

## NON-NEOPLASTIC COLORECTAL POLYPS

### 

#### Hyperplastic polyps

are the most common type of colorectal polyps. They were once considered as a separate entity but they are nowadays believed to be a subgroup of serrated polyps that do not share the malignant potential of the other subgroups, SSAs and TSAs [39]. These polyps are usually smaller than 5 mm in diameter (*diminutive* polyps), sessile, and most commonly found in the distal colon and rectum. They show histological characteristics of hyperplasia without dysplasia; for this reason, they are not considered pre-malignant. Unfortunately, hyperplastic polyps cannot always be distinguished from adenomatous polyps at endoscopy and they are therefore often removed. Hyperplastic polyps greater than 2 cm in diameter may pose a slight risk of dysplasia and malignant degeneration.

#### Hamartomatous polyps

also known as ‘juvenile polyps’, consist mainly of connective tissue (smooth muscle, *lamina propria*, and inflammatory infiltrates) covered by a hypertrophic epithelium. Macroscopically, they are pedunculated, cherry-red, smooth polyps and are sometimes indistinguishable from pedunculated adenomatous polyps. Hamartomas can appear sporadically or as part of a polyposis syndrome. A sporadic hamartomatous polyp is usually solitary and appears at an early age; 75% occur in children younger than ten years (hence the name ‘juvenile’ polyp). Sporadic hamartomatous polyps do not usually harbor any malignant potential. However, because they are highly vascularized, they tend to cause bleeding. Intussusception and obstruction may also occur. Multiple hamartomatous polyps appear with genetic polyposis syndromes that are reviewed in detail below.

#### Inflammatory polyps (pseudopolyps)

most commonly occur in patients with inflammatory bowel disease, mainly ulcerative colitis. They may also occur following an event of infectious or ischemic colitis. These lesions are not true polyps but rather accumulations of inflammatory infiltrations with distorted mucosal anatomy. These lesions are not pre-malignant, but they cannot be distinguished from adenomatous polyps based upon colonoscopic appearance. Therefore, the recommendation is to biopsy them. In general it is not necessary to resect them unless symptomatic. Microscopic examination of inflammatory pseudopolyps shows islands of normal, regenerating mucosa (the polyp) surrounded by areas of mucosal loss. Polyposis may be extensive, especially in patients with severe colitis, and may mimic familial adenomatous polyposis.

#### Submucosal colorectal lesions

both benign and malignant, can be mistaken for colorectal polyps. Such benign lesions include lipomas, isolated lymphoid nodules, pneumatosis cystoides intestinalis, hemangiomas, endometriosis, and others. Malignant or pre-malignant lesions that can be mistakenly identified as polyps are carcinoid tumors, gastro-intestinal stromal tumors (GISTs), lymphomas, metastases and others. It is important to diagnose these lesions and, in cases when the diagnosis is in doubt and a colonoscopic biopsy fails to diagnose the lesion, it is possible to continue the evaluation using computed tomography. In case of a rectal lesion, evaluation with endorectal ultrasonography (ERUS) and ERUS-guided biopsy might be of use.

## POLYPOSIS SYNDROMES

The presence of a systemic process that promotes the development of multiple gastro-intestinal polyps is termed ‘polyposis’. Hereditary gastro-intestinal polyposis syndromes account for approximately 1% of all cases of colorectal cancer and are associated with a broad spectrum of extra-colonic tumors [46]. Early detection and accurate classification of these syndromes are essential in order to initiate a surveillance program for the early detection of cancer. Several polyposis syndromes have been described, and each has its own genetic basis and characteristic polyp distribution, clinical presentation, and malignancy risk. The more prevalent of these syndromes are reviewed here.

### Familial adenomatous polyposis

**Figure 4.**
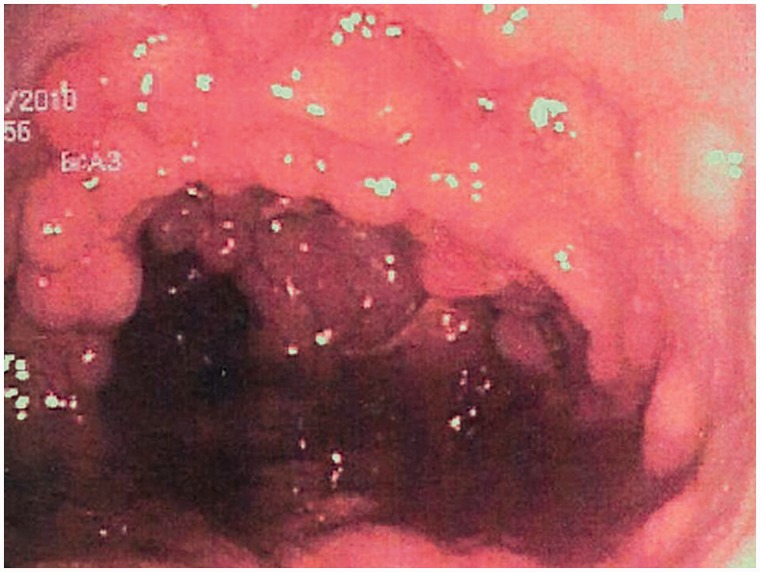
Colonic mucosa carpeted with adenomatous polyps in a patient with familial adenomatous polyposis.

Familial adenomatous polyposis (FAP) is the prototypical hereditary polyposis syndrome. It is an autosomal dominant genetic disease, caused by a mutation in the APC gene on chromosome 5q. Several mutations in this gene have been described: the clinical presentation varies according to the specific mutation. Generally, the clinical presentation of FAP is the development of multiple adenomatous polyps throughout the colon and rectum (Figure 4). In addition, some extra-colonic and extra-intestinal manifestations may occur. The onset is early during childhood or adolescence and the number and size of polyps increase with age. In the severe form of FAP, the patient develops thousands of colonic adenomas by adulthood and the mean age of colorectal cancer development is 35 years if the patient is left untreated. Colorectal cancer can be prevented by identification of the high-risk population and by the timely implementation of rigid screening programs [47].

FAP was first clinically described by Virchow in 1863. Only 64 years later, it was demonstrated that FAP was an hereditary disorder transmitted in an autosomal dominant fashion. Mutations in the 5q chromosome were discovered to be associated with FAP in 1986. Later on, in the mid-1990s, the APC gene was discovered to be the genetic basis of FAP. The APC gene is composed of 2,843 codons in 15 translated exons. Mutations in this gene result in the deactivation of its product, which regulates multiple signaling intracellular pathways by enhancing the activity of glycogen synthase kinase-3, which is essential for many cellular processes [48]. Most of the mutations in the APC gene result in premature stop codons: thus, the gene product is not complete. Mutations in the first three exons result in an attenuated clinical syndrome due to downstream ribosomal re-entry site. In contrast, downstream mutations—especially in exon 15—result in a virulent form of the disease. This exon, which is the largest in the gene, is also the site of the majority of acquired mutations in sporadic colorectal cancers (as well as in the other allele of FAP patients). It is likely that severity of the phenotype is influenced not solely by the specific APC gene mutation and that other factors, such as co-occurrent gene variants are also important for determining the severity of disease and the presence of extra colonic manifestations. Historically, a few other genetic polyposis syndromes were described as different entities from FAP. These presented the gastro-intestinal features of FAP, combined with extra-intestinal manifestations, and include mainly Gardner's syndrome (colonic polyps, epidermal inclusion cysts, osteomas) and Turcot's syndrome (colonic polyps and brain tumors). Nowadays they are considered to be a part of the FAP variety, resulting from different germline mutations in the APC gene and, as described above, possibly from co-occurring mutations in other genes that have not yet been identified.

### Gastro-intestinal features of FAP

The most common manifestation of FAP is the invariable presence of multiple colonic polyps (Figure 4). As mentioned above, the number of polyps is usually in the thousands and, in such patients, the natural development of colorectal cancer occurs at the age of approximately 35–40. In attenuated forms of the syndrome, which arise from different germline mutations in the APC gene, 20–100 polyps may be dispersed throughout the colon. The natural course of cancer development in these patients occurs at about the age of 50–60 years. Extra-colonic manifestations may include gastric, duodenal, and peri-ampullary polyps. Most of the gastric polyps represent fundic gland hyperplasia and have a low malignant potential. Gastric adenomas, albeit rare in the context of FAP, are recognized occasionally in FAP patients. Duodenal (mainly peri-ampullary) polyps, however, are adenomatous in nature and therefore should be considered pre-malignant. Compared with the colonic polyps, these lesions tend to appear at a later stage of life and usually are not as crowded. The lifetime risk of an FAP patient developing peri-ampullary carcinoma is estimated at 5–10%. Polyps and cancer have also been found in the jejunum and ileum of patients with FAP, although at a much lower incidence.

### Extra-intestinal manifestations

FAP patients commonly present some extra-intestinal manifestations. Approximately 75% of FAP-affected individuals have congenital hypertrophy of the retinal pigmented epithelium (CHRPE), which can be detected by ophthalmoscopy. This abnormality is not specific to FAP; however, large multiple bilateral lesions are diagnostic of the disease. Osteomas are benign bone tumors that usually present as visible and palpable prominences in the skull, mandible, and tibia. On radiographs, they appear as hypodense lesions. Other radiographic abnormalities in FAP include impacted or supernumerary teeth.

**Figure 5.**
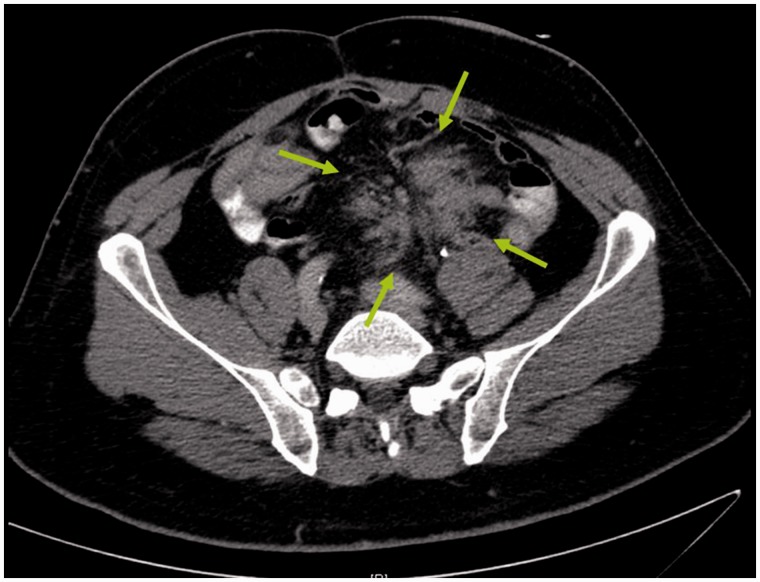
Image from a computed tomography of a desmoid tumor in a patient with familial adenomatous polyposis. The tumor is marked by the arrows. The tumor has developed many years after staged total proctocolectomy with end ileostomy. A surgical attempt to resect the tumor was undertaken, without success.

Desmoid tumors develop in 10–15% of patients with FAP. These are locally invasive tumors of the retroperitoneum and the abdominal wall (Figure 5). After surgical procedures, dense fibrous tissue forms within the abdominal cavity in some patients with FAP. This fibrous tissue tends to aggressively invade the mesentery or retroperitoneal tissues and form tumors. Although these tumors seldom metastasize, they are often locally invasive; direct invasion of retroperitoneal organs, such as the great vessels or ureters or to the bowel wall, might result in patient death. If the mesentery is involved, the intestine might be tethered and bowel obstruction might result. Desmoid tumors tend to appear most often after abdominal surgery but they may also appear spontaneously. They are the second-greatest cause of death after colorectal cancer in FAP patients. Five-year survival of patients with abdominal desmoid tumors varies, depending on tumor stage, size, and other factors; this outcome has been reported to be as low as 53% [49]. Patients with FAP are also at increased risk of other malignancies. These include cancers of the liver, extra-hepatic biliary tree, adrenals, and thyroid. The historic Turcot's syndrome is characterized by colonic polyposis and brain tumors.

### Diagnosis and genetic testing of FAP

Clinical diagnosis of FAP is obvious and is confirmed by the appearance of multiple colorectal polyps in family members of affected patients who undergo screening colonoscopy, or by the same clinical picture in patients who were not known to be a relative of an FAP patient. When patients are clinically diagnosed with FAP, they should undergo germline mutation genetic diagnosis, in order to counsel their first-degree relatives regarding screening and treatment. Commercially available genetic tests will detect approximately 80% of the APC gene mutations and, if such a mutation is found in a diagnosed patient, it is feasible to diagnose the disease using the same test in first-degree relatives. The risk of mutation inheritance is 50%, as in any other autosomal dominant disorder. Hence, the use of genetic testing enables the concession of screening programs in 50% of offspring and other first-degree relatives of patients with FAP. Accordingly, if the analysis of a single blood test demonstrates non-inheritance of a mutated APC gene, the individual can avoid yearly endoscopic screening and should require only occasional colonoscopy.

In cases when no mutation is found in the patient known to have FAP, or if genetic testing was not performed, screening sigmoidoscopy is recommended for detecting FAP mutation carriers among a diagnosed patient's first-degree relatives. These individuals should undergo sigmoidoscopy at age 10–12 years and then annually until age 35; after age 35, if they have not been diagnosed up to that point, they should undergo sigmoidoscopy at 3-year intervals. Upper gastro-intestinal endoscopy should be performed every 1–3 years, starting when colorectal polyps are first identified. The same screening program should apply to relatives of known patients with FAP who test positive for the APC gene mutation.

### Treatment of FAP

The treatment of FAP is prophylactic surgery, directed at removal of all affected colonic and rectal mucosa. Total proctocolectomy with the formation of an ileal pouch and ileoanal anastomosis (TPC-IPAA), also known as ‘restorative proctocolectomy’, is currently the most commonly recommended operation. The same procedure is performed for medically refractory ulcerative colitis; however, the functional results are better for patients with FAP. The overall quality of life for patients with FAP undergoing TPC-IPAA is comparable with that of the general population [50]. Nevertheless, due to its pelvic maneuvers, this procedure involves risks of autonomic nerve injury, sexual dysfunction and infertility [51]. These risks can be prevented by close rectal wall dissection and a laparoscopic approach [52]. The laparoscopic approach, which gives a better cosmetic result, has proven to be superior to laparotomy [53]. Further advantages of laparoscopic TPC-IPAA are associated with less adhesion formation, which reduces risks of small bowel obstruction. The recommended timing for surgery is soon enough to prevent malignancy but, if possible, late enough for the individual to reach physical and psychological adulthood.

An alternative surgical approach is total abdominal colectomy with ileo-rectal anastomosis (TAC-IRA). This approach is considered for patients with FAP who have a small rectal polyp burden. For several reasons, this operation is technically simpler and has fewer potential complications. Firstly, the pelvic dissection is avoided; hence, potential autonomic nerve injury—that could result in impotence in males and infertility in females—is prevented. Secondly, the anastomotic leakage rate is lower following ileo-rectal anastomosis than after pouch-anal anastomosis [54]. Obviously, the disadvantage of TAC-IRA is the need for frequent surveillance proctoscopy (every 6–12 months) with removal of polyps and the increased risk of rectal cancer following surgery, which is 4% at 5 years, 8% at 15 years, and 25% at 20 years [54, 55]. Sulindac and celecoxib were shown to produce partial regression of polyps; these are feasible treatment options for patients who undergo TAC-IRA [56, 57]. Nevertheless, these patients will always need surveillance proctoscopy and, potentially (33% lifelong), completion proctectomy with end ileostomy or IPAA. The incidences of polyp reappearance and cancer development are higher when the drug is stopped.

As discussed earlier, patients with FAP have an increased risk of duodenal and ampullary polyps, which require attention. Approximately 90% of patients with FAP will develop peri-ampullary polyps during their lifetime, but only 10–20% will develop duodenal cancer. A surveillance upper gastro-intestinal endoscopy should be performed periodically, as discussed above. Endoscopic polypectomy, if possible, should be performed. If endoscopic polypectomy is not feasible—or, in the case of ampullary cancer, discovered at an early stage—transduodenal wide local excision or even pancreato-duodenectomy (Whipple procedure) is indicated. In contrast to lower gastro-intestinal malignancy, no drug has been proven effective in chemoprevention of upper gastro-intestinal malignancy in patients with FAP.

Abdominal desmoid tumors can be a challenging extra-intestinal manifestation of FAP. Small desmoid tumors confined to the abdominal wall can be successfully resected. In contrast, the surgical treatment of mesenteric desmoids is dangerous and should generally be undertaken by a skilled, small-bowel transplant team. In such a case, initial treatment usually consists of sulindac or tamoxifen because desmoid tumors might be hormonally responsive; however, success is rare [58]. Other treatment options are radiation therapy in cases of relatively superficial tumors, cytotoxic chemotherapy with doxorubicin and, in cases of c-Kit gene mutations, biologic treatment with imatinib (Gleevec).

### MUTYH-associated polyposis syndrome

MUTYH-associated polyposis (MAP) is an autosomal recessive polyposis syndrome that was first identified in 2002. It is caused by bi-allelic mutations in the MUTYH gene, which is a DNA glycolase responsible for base excision repair [59, 60]. Individuals carrying two copies of the mutation have a significantly increased risk of polyposis and colorectal cancer. The polyps seen in MAP are typically small tubular or tubulo-villous adenomas or hyperplastic polyps. In untreated patients, the cumulative risk of colorectal cancer is estimated as 80% by age 70 years. In the characteristic phenotype, the patient develops tens to hundreds of colorectal polyps, which are detected at adulthood; this contrasts with the thousands of polyps that develop throughout childhood in patients with FAP. Hence, MAP is very similar phenotypically to attenuated forms of FAP. Upper gastro-intestinal polyps and additional extra-intestinal features commonly seen in FAP may characterize MAP, but they are much less common. Approximately 1–2% of the general population carries a mutation in the MUTYH gene [59]. The parents of a bi-allelic carrier are obligate carriers of MUTYH mutations. Questions remain regarding the medical significance of carrying one copy of a MUTYH mutation and whether it may significantly increase risks of colorectal cancer.

Pathogenesis of MAP is presumed to be the accumulation of acquired mutations in the APC gene, due to loss of MUTYH DNA base excision repair activity. Genetic testing for MUTYH mutation is complicated by the phenotypic overlap of MAP with FAP. Therefore, genetic testing is performed mainly in cases of suspected FAP when no mutations in the APC gene are found, or when a patient with the clinical presentation of FAP has no relevant family history. Once diagnosed, MAP patients should be monitored for the development of colorectal cancer. Patients should undergo full colonoscopy starting at age 25–30 years and then every 3–5 years if no polyps are detected or more frequently if they are. With the patient's diagnosis, siblings should be genetically tested and counseled.

### Peutz-Jeghers syndrome

Peutz-Jeghers syndrome is a familial autosomal dominant syndrome characterized by polyposis of the small intestine and, to a lesser extent, polyposis of the colon and rectum. It was first described by Hutchinson in 1896. In the 1940s, separate descriptions by Peutz and then Jeghers gave the disease its name. The gene responsible for the phenotypic appearance of Peutz-Jeghers syndrome is called STK11 and is located at chromosome 19p. This is a tumor suppressor gene that encodes a serine /threonine kinase (hence its name). Mono-allelic loss of this gene in Peutz-Jeghers syndrome was recently shown to be associated with diverse methylation patterns in colonic crypts and hence to provoke the evolution of pre-malignant lesions at an accelerated pace, compared with that observed in the general population [61]. The polyps of Peutz-Jeghers syndrome are non-neoplastic hamartomas, consisting of connective tissue and smooth muscle, covered by hyperplastic epithelium. They appear throughout the gastro-intestinal tract and are mostly scattered in the small bowel. A common clinical presentation is of gastro-intestinal bleeding or intestinal obstruction (from intussusception) [62].

Peutz-Jeghers syndrome is also associated with extra-intestinal manifestations: characteristic skin and dark bluish buccal mucosal pigmentation is often found in patients with Peutz-Jeghers. Common places for the skin lesions are the lips, hands, feet, genitalia, and anus. These skin lesions typically appear at birth and, by puberty, they tend to become lighter, in contrast to regular freckles which appear only at childhood. Patients are also at increased risk for the development of various early-onset neoplastic diseases, including cancer of the gastro-intestinal tract, breast, ovary, cervix, fallopian tubes, thyroid, lung, gall bladder, bile ducts, pancreas, and testicles. One study of 419 cases of Peutz-Jeghers syndrome showed 96 cancers in 85 patients (11 patients had 2 different primary cancers) [63]. The most common cancers were colorectal (17 cases), breast (16 cases) and pancreatic (9 cases). Among Peutz-Jeghers patients, the cumulative risk of developing any cancer by ages 20, 30, 40, 50, 60, and 70 years was calculated as 2%, 5%, 17%, 31%, 60%, and 85%, respectively.

The clinical picture of gastro-intestinal hamartomatous polyposis and of characteristic cutaneous and mucosal pigmentation provides the diagnosis of Peutz-Jeghers syndrome. Endoscopic removal of a polyp and its histological examination can also provide the diagnosis. In cases of a known family member who has Peutz-Jeghers syndrome, genetic screening is feasible. The recommended screening regimen for patients who are known to have Peutz-Jeghers syndrome consists of colonoscopy, upper endoscopy and small bowel follow-through, beginning at age 20 years and repeated at 2-year intervals. In addition, patients should be screened periodically for other malignancies: breast (physical examination and mammography annually, starting at age 25 years); cervix and ovary (physical exam, transvaginal ultrasound, and Pap smear annually), and testicles (physical examinationand ultrasonography as needed) [62].

Because the entire length of the gastro-intestinal tract may be affected, surgery is reserved for symptoms such as obstruction or bleeding or for a diagnosed cancer. If surgery is performed, an attempt should be made to remove as many polyps as possible with the aid of intra-operative endoscopy and polypectomy. Any polyp larger than 1.5 cm should be removed if possible. Another indication for bowel resection in Peutz-Jeghers syndrome is for patients in whom polyps develop adenomatous features. Usually, this is not evident until cancer is present.

### Familial juvenile polyposis

Juvenile polyps are hamartomatous benign polyps composed of fluid and mucous-filled spaces within the *lamina propria*. As their name implies, they appear at childhood and may cause bleeding or intussusception. For these reasons, once recognized they should be treated by endoscopic removal. Familial juvenile polyposis is an autosomal dominant disorder in which patients develop hundreds of juvenile polyps in the colon and rectum. Polyps may arise in all other parts of the gastro-intestinal tract as well. Unlike solitary juvenile polyps, these lesions may harbor some extent of adenomatous characteristics and degenerate into adenomas and subsequently carcinoma. The genetic basis of familial juvenile polyposis is not fully known. Mutations in the tumor suppressor gene SMAD4 on chromosome 10q are believed to cause up to 50% of cases. Another gene that has been reported to be involved is BMPR1A [64–66]. Both of these genes are involved in the TGFβ signaling pathway [66]. The genetic mutations have not been identified in all cases of familial juvenile polyposis. The classical clinical presentation is of lower gastro-intestinal bleeding, which leads to colonoscopy. Bowel obstruction from large polyps or from intussusception is not rare, and passage of auto-amputated polyps may also occur. Protein-losing enteropathy may develop. The endoscopic appearance of multiple polyps and their histological evaluation can make the diagnosis, which is considered in any patient who has (i) at least 3–5 juvenile polyps of the colon or (ii) multiple juvenile polyps found throughout the GI tract or (iii) any number of juvenile polyps if there is a family history of juvenile polyposis syndrome [67]. Screening should begin at age 15 years if the patient is asymptomatic or earlier if symptoms commence, and should be repeated annually if polyps are revealed, or every 2–3 years if no polyps are seen. Some of the patients with SMAD4 mutations present with another autosomal dominant syndrome, hereditary hemorrhagic telangiectasia (HHT) in which bleeding from arteriovenous malformations (AVMs) may occur. Hence, juvenile polyposis patients should also have periodic screening for the presence of gastro-intestinal and pulmonary AVMs [67, 68].

Congenital abnormalities are present in 20% of the patients, including malrotation, hydrocephalus, cardiac lesions, Meckel's diverticulum, and mesenteric lymphangioma. The risk of malignancy is mainly in the colon and rectum, and the lifetime risk of colorectal cancer in juvenile polyposis patients has been stated to be as high as 39% [65–66]. There is also an increased risk for other cancers, mainly gastric, duodenal, and pancreatic.

As in FAP, treatment for juvenile polyps is prophylactic surgery, due to the observation that up to 39% of these patients will develop cancer. The type of surgery recommended depends upon the degree of rectal involvement. If the rectum is relatively spared, TAC-IRA is a feasible option, with subsequent close surveillance of the retained rectum. One recent study has shown most polyps in this syndrome to be located at the right colon [65], so the TAC-IRA option should be realistic for the majority of patients. If the rectum is heavily involved, TPC-IPAA is the more appropriate operation. In case of a large polyp burden in other parts of the GI tract, other surgeries may be warranted, i.e. gastrectomy, small bowel resection, etc.

### Serrated polyposis syndrome

Serrated polyposis syndrome (SPS), also known as ‘hyperplastic polyposis syndrome’, is a newly recognized, usually sporadic, and rare condition for which the molecular etiology and pattern of inheritance (if any) remain unknown [39, 69]. The diagnosis of SPS is based on one of the following colonoscopic findings: (i) at least five histologically confirmed serrated polyps proximal to the sigmoid, with at least two of these being greater than 1 cm; (ii) any number of serrated polyps proximal to the sigmoid and a first-degree relative with SPS or (iii) more than 20 serrated polyps scattered throughout the colon [1, 70].

Metrics regarding increased colorectal cancer risk in SPS vary from zero to 69%, according to various studies, although the data are likely to be biased [1,70]. A recent study has demonstrated the development of colorectal cancer in 35% of patients with SPS, with most cases detected in their primary colonoscopy [71]. Cancer was shown to develop in serrated polyps as small as 4 mm and the cumulative risk was 7% at 5 years. The presence of cancer was significantly associated with an increasing number of hyperplastic polyps and the presence of serrated adenomas [71]. Another recent study has demonstrated three different, but overlapping, clinical phenotypes within SPS: (i) relatively few large right-sided polyps, (ii) many small left-sided polyps and (iii) a combination of both left- and right-sided polyps. The right-sided phenotype had more sessile serrated polyps and tended to develop into colorectal cancer in patients at a younger age [72]. These observations support the ‘serrated adenoma pathway’ theory, which states that serrated adenomas are associated defects in DNA repair mechanisms and, hence, with a faster adenoma-to-carcinoma sequence and the development of colorectal cancer [39, 41, 42]. Thus, in order to prevent malignancy in SPS, it is advisable to detect and remove all polyps. If this is not feasible, surgical resection should be considered [71].

### PTEN hamartomatous tumor syndromes

***Cowden's syndrome*** is an autosomal dominant disorder with hamartomas of all three embryonic cell layers. The affected gene is PTEN on chromosome 10q. Approximately 80% of patients harbor a germline mutation in this tumor suppressor gene [73]. Gastro-intestinal polyps—most commonly of the colon and stomach—are typical of the syndrome but are usually asymptomatic. Extra-intestinal manifestations include mucocutaneous lesions, thyroid adenomas and goiter, fibro-adenomas and fibrocystic disease of the breast, uterine leiomyomas, and macrocephaly. In addition to the risk of colorectal cancer, about which there is not much data, patients have a 10% risk for thyroid cancer and up to a 50% risk for invasive carcinoma of the breast; therefore, patients with Cowden's syndrome should be screened for the development of various cancers. Annual physical examinations should be performed with special attention to thyroid and breast. Mammography should be performed annually, starting at age 30 years or at an age 5 years younger than the earliest breast cancer case in the family.

Another rare type of familial syndrome due to a mutation in the PTEN gene is Bannayan-Riley-Ruvalcaba Syndrome, an autosomal dominant syndrome with features of hamartomatous colonic and ileal polyps, as well as other manifestations. Lhermitte-Duclos Disease is another variant of Cowden Syndrome, which is associated with cerebellar hamartomatous overgrowth [74]. These three syndromes are sometimes referred to as ‘PTEN hamartomatous tumor syndromes’ (PHTS) [70].

There are no specific guidelines regarding screening colonoscopy in either of the syndromes because the chance of malignant degeneration of colonic polyps is not known and is thought to be low. A recent study reports on 10 Cowden Syndrome patients who underwent a mean of 2.4 colonoscopies each, starting at an average age of 31.7 years. Eight patients were found to have colonic polyps, mostly hyperplastic and hamartomatous, but also adenomatous (three patients). One patient was diagnosed with rectal cancer [75].

Treatment of PHTS is based upon symptoms, and prophylactic colorectal surgery is not recommended.

### Hereditary mixed polyposis syndrome

Hereditary mixed polyposis syndrome (HMPS) is a syndrome in which patients present with multiple colorectal polyps of different histopathological types (adenomatous, hyperplastic and hamartomatous), with an autosomal dominant pattern of inheritance, but not fulfilling diagnostic criteria for any of the other polyposis syndromes [76]. Data are lacking, regarding the tendency of HMPS patients to develop colorectal cancer, but it is believed to be higher than in the general population [76]. The gene responsible for this syndrome has not been identified but SMAD4*,* BMPR1A and others have been suggested [76–78].

### Cronkite-Canada syndrome

Cronkite-Canada syndrome is an acquired, non-familial disorder, in which patients develop gastro-intestinal polyposis in association with alopecia, cutaneous pigmentation, and onycholysis (atrophy of the fingernails and toenails). Diarrhea is a prominent symptom, and vomiting, malabsorption and protein-losing enteropathy may occur. In fact, the polyposis is attributed to mucosal and submucosal diffuse inflammation, which is believed to be the factor that causes the diarrhea. On histological evaluation, the polyps resemble juvenile polyps. Occasionally, malignant degeneration of a polyp occurs, but most patients die of the protein-losing enteropathy despite maximal medical therapy. Surgery is reserved for complications of polyposis, such as obstruction.

## CONCLUSIONS

Colorectal polyps are a common finding in screening colonoscopies. Most of these are of no clinical significance but, due to their high prevalence, the minority of such polyps that bear a malignant potential still represents a central issue in preventive medicine. Early identification and removal of these lesions is a highly effective method of preventing morbidity and mortality from colorectal carcinoma. The diagnosis of a colorectal polyposis syndrome is initially suggested, based on colonoscopic findings and polyp histology. Because different syndromes can resemble each other phenotypically, molecular genetic studies are essential for final diagnosis, cancer risk assessment, and decision-making regarding a surveillance program and treatment. In addition, identification of the familial mutation in an affected patient is a prerequisite for future testing of asymptomatic relatives.

**Conflict of interest:** none declared
